# Resonance-Enhanced Multiphoton Ionization Studies of the Lower Electronically Excited States of Flavone

**DOI:** 10.1021/acs.jpca.3c00202

**Published:** 2023-02-13

**Authors:** Jiayun Fan, Wybren Jan Buma

**Affiliations:** † Van’t Hoff Institute for Molecular Sciences, University of Amsterdam, Science Park 904, 1098 XH Amsterdam, The Netherlands; ‡ Institute for Molecules and Materials, FELIX Laboratory, Radboud University, Toernooiveld 7c, 6525 ED Nijmegen, The Netherlands

## Abstract

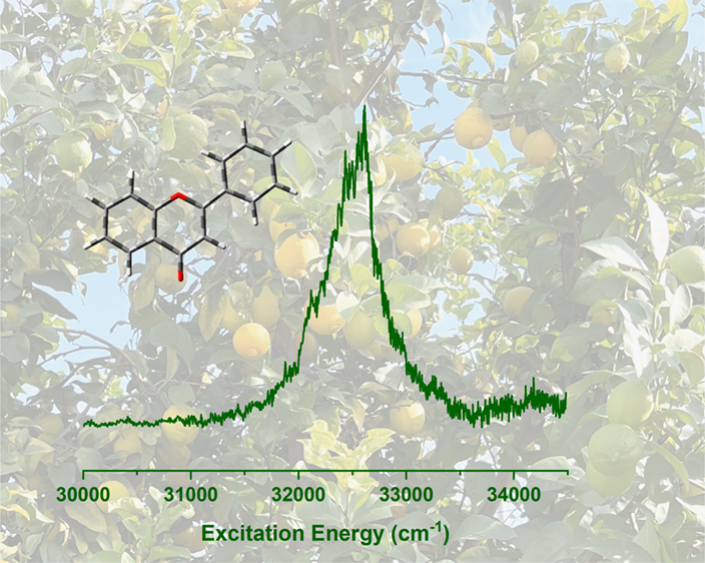

The spectroscopic and dynamics properties of flavone—the core chromophore of a wide variety of naturally occurring ultraviolet protecting filters—have been studied under supersonic beam conditions using (1 + 1′) resonance-enhanced two-photon ionization spectroscopic techniques. Excitation spectra recorded under such conditions are found to differ significantly from previously reported spectra. Pump–probe studies find that intersystem crossing is the dominant decay pathway of the excited singlet manifold, in agreement with previous solution phase studies and quantum chemical predictions for the isolated molecule. Microsolvation studies on flavone–water clusters reveal that the addition of one and two water molecules leads to considerable shifts in excitation energies but that further complexation does not result in further noticeable shifts. The relaxation pathways of the electronically excited states, on the other hand, do not appear to be influenced by interactions with the solvent molecules. Finally, photoionization spectra have enabled the accurate determination of the adiabatic ionization energy to the ground state of the molecular ion—key to the antioxidant properties of flavone—as 65,415 cm^–1^ (8.110 eV).

## Introduction

Flavonoids are a class of natural compounds characterized by a backbone consisting of two phenyl rings connected by a heterocyclic pyran ring. As yet, more than 8000 flavonoids have been identified in nature that differ by the substituents on these rings.^1−3^ Among the different classes of flavonoids, flavone derivatives play a particularly prominent role because of their protective properties against oxidative processes originating from free radical species or absorption of ultraviolet (UV) radiation.^4−8^ Concurrently, they have found widespread medical applications because of their anti-viral, anti-tumor, and anti-inflammatory activities as well as neuro- and cardioprotective properties,^9−14^ to name only a few of the many positive pharmacological effects identified so far.

The photoprotective properties of flavones derive from their strong absorption of light in the UV-B region.^7,15−17^ Because of this strong absorption, flavones have attracted considerable interest for applications such as UV filters in commercial sunscreen formulations.^8,18,19^ In particular, the 3- and 5-hydroxy-substituted derivatives have from this point of view been of interest as it has been shown that in these compounds the photon energy can be dissipated into harmless heat efficiently and on an ultrafast timescale by excited-state intramolecular proton transfer.^20−25^ In order to tailor the photoprotective properties of flavone-based compounds, significant effort is presently dedicated to obtain a fundamental understanding of the influence of substituents and environment on the spectroscopic and dynamic properties of the electronically excited states involved in absorption and dissipation of the photon energy.

**Figure 1:**
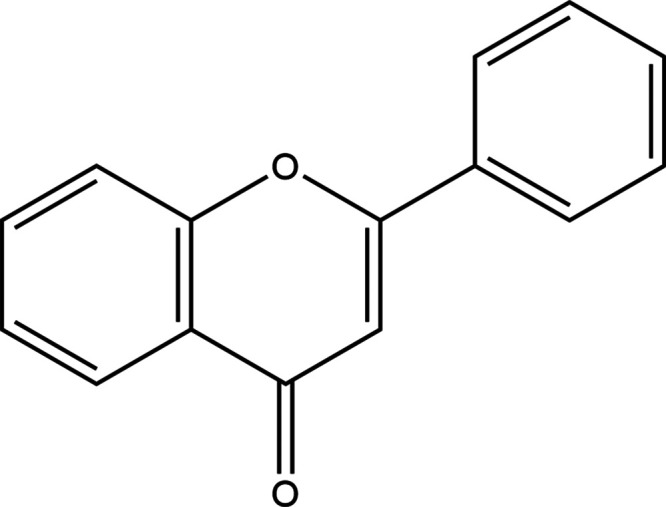
Molecular structure flavone.

The starting point for such considerations is the electronic manifold of flavone, the core chromophore of such compounds (Figure 1). Previous experimental solution studies combined with theoretical calculations have led to the conclusion that the lowest electronically excited singlet state of flavone is a ^1^nπ* state with negligible oscillator strength, while at higher excitation energies a strong UV absorption is observed that has been assigned to two close-lying ^1^ππ* states.^26−30^ Photoexcitation of these states has been shown to be followed by a very efficient intersystem crossing (ISC) process that populates on a picosecond timescale the triplet manifold with a nearly unit quantum yield.^31,32^ Further detailed insight into these processes has been obtained from high-level density functional theory and multireference configuration interaction (DFT/MRCI) calculations that determined vertical and adiabatic excitation energies of electronically excited singlet and triplet states, as well as spin–orbit coupling matrix elements between singlet and triplet states.^28,29^

Although flavone has been studied extensively under solvated conditions, studies of the compound under isolated conditions are notoriously lacking. In fact, the only report of an excitation spectrum recorded under such conditions has been provided in the transient absorption studies of ref (29) in which a resonance-enhanced two-photon ionization (R2PI) spectrum of jet-cooled flavone was shown. This spectrum displayed two broad features that were concluded to be consistent with the predicted excitation energy of the S_2_(ππ*) state. Nevertheless, when these features are considered in more detail, one rapidly comes to the conclusion that their assignment is still far from clear. In order to provide further insight into the spectroscopic and dynamic properties of flavone, we have therefore performed similar R2PI studies of the excitation spectrum of flavone in combination with ns pump–probe measurements. In contrast to the previous studies, however, we have not used the same laser for excitation and ionization but have adopted a two-color scheme that employs an ArF excimer laser (193 nm) for ionization to ensure that ionization indeed takes place via a (1 + 1′) ionization process. For practical applications, it is important to determine how the properties of the compound under isolated conditions are modified by its interactions with the environment. Analogous studies have therefore been performed on complexes of flavone with a varying number of water molecules. Finally, flavone is also well-known and used for its antioxidant properties as has been mentioned above. A key parameter determining these properties is the adiabatic ionization energy of the compound. As yet, only low-resolution He(I) photoelectron spectra have been reported.^33,34^ In order to improve on these energies, photoionization spectra have therefore been recorded that indeed have allowed for a significantly more accurate value of the adiabatic ionization energy.

## Experimental and Theoretical Methods

Flavone was purchased from Sigma-Aldrich chemicals and used without any purification. R2PI experiments have been performed employing a molecular beam setup described previously.^35^ (see also Supporting Information SI1). Briefly, flavone was heated up to 150 °C within a glass container in order to obtain sufficient vapor pressure and expanded using neon at 1.5 bar as a carrier gas through a General Valve pulsed nozzle with a 0.5 mm orifice diameter, which was kept 5 °C higher than the main body in order to avoid clogging. After being skimmed by a 2 mm skimmer, the molecular beam entered an ionization chamber where ions or electrons were detected using either a reflection time-of-flight (R.M. Jordan Co.) setup for mass-resolved ion detection or a custom-built setup (R.M. Jordan Co.) for electron detection.

(1 + 1′) R2PI excitation spectra were recorded using a frequency-doubled Sirah Cobra-Stretch dye laser operating on DCM/Pyrromethene 597 pumped by a Spectra-Physics Lab 190 Nd/YAG laser for excitation and a Neweks PSX-501 ArF excimer laser (193 nm, 6.42 eV) for ionization. Typically, pulse energies of 10–50 μJ and 1 mJ were used for excitation and ionization, respectively. For recording ionization threshold spectra, the same pulsed dye laser system has been used in combination with another pulsed dye laser system consisting of a frequency-doubled Sirah Precision Scan dye laser operating on DCM or Pyrromethene 597 and pumped by a Spectra Physics Lab 190 Nd/YAG laser. In these experiments, typical pulse energies were employed of 1 mJ for the pump laser and 2–4 mJ for the probe laser.

In order to analyze the observed electronic transitions and to determine the ionization energy of flavone and its complexes, DFT has been used to determine the equilibrium geometries and harmonic force fields of flavone in the electronic ground state of the neutral (S_0_) and cation (D_0_), while time-dependent DFT (TD-DFT) was employed to optimize the geometry of the molecule in the first three electronically excited singlet states of the neutral and to determine the associated harmonic force fields. Such calculations have been performed at both the B3LYP/TVZP and wB97XD/cc-pVDZ level.^36−39^ For comparison with the experimental results, the obtained equilibrium geometries and force fields were used to obtain Franck–Condon spectra at wB97XD/cc-pVDZ level for S_*n*_ ← S_0_ transitions for which vibrational frequencies were scaled using a scaling factor of 0.953.^40,41^ All calculations have been performed with the Gaussian16, Rev.C.01 suite of programs.^42^

## Results and Discussion

**Figure 2:**
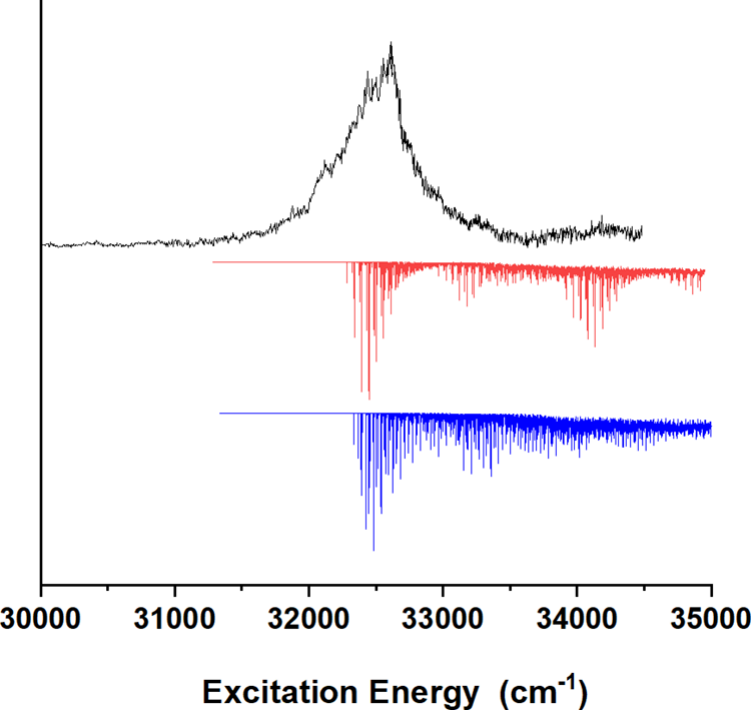
Experimental 1 + 1’ R2PI excitation spectrum of flavone (black curve). The red and blue traces depict stick spectra of Franck–Condon simulations of the excitation spectra of the S_2_(ππ*) and S_3_(ππ*) states, respectively, obtained at the wB97XD/cc-pVDZ level and employing a scaling factor for vibrational frequencies of 0.953.

The black curve in Figure 2 displays the (1 + 1′) R2PI excitation spectrum of flavone seeded in a molecular beam in the UV-B region of 30,500–34,500 cm^–1^ (327.9–289.9 nm). Importantly, no further bands could be detected below 30,500 cm^–1^ or at higher excitation energies up to 35,000 cm^–1^. This spectrum is dominated by a broadband with a maximum at about 32,560 cm^–1^ (∼307 nm) and width on the order of 1000 cm^–1^. As the band does not have a Lorentzian profile, it has to be concluded that it consists of overlapping vibronic bands that have become unresolved because of extensive Franck–Condon activity of low-frequency modes and/or lifetime broadening as is indeed confirmed by our quantum chemical calculations (vide infra). The onset of the band suggests that the vibrationless transition to the pertaining electronic state is located between 31,200 and 31,600 cm^–1^. Although the band does not display a well-resolved substructure, the low-energy side of the band appears to display a vibrational progression of a mode with a frequency of about 400 cm^–1^. At higher excitation energies, the spectrum displays a weak activity in the region around 34,000 cm^–1^ where the activity of C=C and C=O stretch vibrations might be expected.

The spectrum shown in Figure 2 deviates significantly from the one-color REMPI excitation spectrum reported previously which showed two structureless bands at 31,104 cm^–1^ (321.5 nm) and 31,397 cm^–1^ (318.5 nm).^29^ These two bands are not observed in the present experiments. Ionization threshold spectra that will be discussed below show that the adiabatic ionization energy to the ground state D_0_ of the ion is 65,415 cm^–1^. This implies that in the one-color REMPI experiments, ionization can only take place at the previously reported energies by a three-photon ionization process, in contrast to the present experiments in which the energy of the ionizing 193 nm photon is large enough to allow for (1 + 1′) ionization. The differences between the two spectra thus lead to the conclusion that the bands that are observed in the one-color spectra should be attributed to resonance enhancement at the two-photon level, that is, the ionization process should be described as a (2 + 1) ionization process. In view of the associated two-photon excitation energies the states that are then responsible for the resonance enhancement are most probably Rydberg states.

Previous DFT/MRCI calculations at the B3LYP/TZVP level indicate that the lowest excited singlet state of flavone is an nπ* state with a negligible oscillator strength for the S_0_ → S_1_ transition, while S_2_ is a ππ* state with a significant oscillator strength in agreement with absorption spectra observed in solution.^29^ Interestingly, these calculations also indicate that at slightly higher excitation energies the transition to the second ππ* state should be located with an oscillator strength that is even larger than that of the transition to S_2_. We have performed additional TD-DFT calculations of vertical and adiabatic excitation energies of the lower-lying electronically excited singlet states using the dispersion-corrected wB97XD functional and the cc-PVDZ basis set. Comparison with TD-DFT calculations at the B3LYP/TZVP level and the results of the previously reported DFT/MRCI calculations (Table 1) shows that one would indeed expect both vertically as well as adiabatically two nearby-lying ππ* states with similar oscillator strengths. The observation that our (1 + 1′) REMPI experiments appear to give evidence only for a single electronically excited state might indicate that the observed broadband at ∼307 nm actually consists of the overlapping transitions to the first and second electronically excited ππ* state, and thereby account for the asymmetric line shape of the band. Alternatively—and assuming that this band is associated with the transition to the first ππ* state—one would need to conclude that transitions to the second ππ* state are too weak to be observed. In view of the predicted oscillator strengths, this would imply, however, that this state is subject to ultrafast internal conversion processes to electronic states with a significantly reduced ionization cross-section. Femtosecond time-resolved ion yield experiments^43,44^ could in this respect be useful to resolve this issue.

**Table 1:** Vertical and Adiabatic Energies (eV) of the Lower Electronically Excited Singlet States of Flavone Calculated at the wB97XD/cc-PVDZ and B3LYP/TVZP Levels, Respectively, with Oscillator Strengths Given in Parentheses (See Supporting Information SI5 for Geometries)

transitions	wB97XD/cc-PVDZ		B3LYP/TVZP	
	vertical	adiabatic	vertical	adiabatic
S_1_ ← S_0_	3.96 (0.0006)	3.70 (0)	3.54 (0.001)	3.06 (0)
S_2_ ← S_0_	4.75 (0.2991)	4.39 (0.4809)	4.19 (0.1448)	3.65 (0.0822)
S_3_ ← S_0_	4.92 (0.2128)	4.66 (0.4387)	4.46 (0.4078)	4.06 (0.6062)

In the DFT/MRCI study, it was found that the molecule adopts a geometry in the ground state in which the benzopyrone and phenyl planes are twisted by ca. 28°. Upon excitation to the electronically excited S_1_, S_2_, and T_1_ states, on the other hand, the molecule became nearly planar.^28^ Based on such a large geometry change extensive Franck–Condon progressions in the low-frequency vibrational modes in which this torsional mode is involved are to be expected. It is important to notice that on top of extensive Franck–Condon activity due to these large geometry changes, also significant lifetime broadening because of ultrafast internal conversion of the excited S_2_(ππ*) state to the lower-lying S_1_(nπ*) state is expected. In this respect, the rate of (170 fs)^−1^ leading to linewidths of 32 cm^–1^ as measured in the gas phase for S_1_(nπ*) ← S_2_(ππ*) internal conversion in trans-azobenzene, a similarly-sized compound with a similar S_1_-S_2_ energy gap, is indicative.^45^

In order to determine whether such progressions might indeed account for the experimentally observed band profile and to investigate to what extent predicted Franck–Condon spectra can resolve whether this band is associated with the S_2_(ππ*) ← S_0_ and/or S_3_(ππ*) ← S_0_ transitions we have calculated such spectra for these two transitions. These wB97XD/cc-PVDZ calculations confirm the previously reported geometry characteristics as a twisting angle of 21° is found for S_0_ while in the two electronically excited states the molecule becomes nearly planar, and give rise to S_2_(ππ*) ← S_0_ and S_3_(ππ*) ← S_0_ Franck–Condon spectra depicted in Figure 2. Following our expectations, these spectra show the extensive activity of low-frequency vibrational modes, in particular of an out-of-plane butterfly mode with a frequency of 33 cm^–1^ and an out-of-plane torsional mode with a frequency of 55 cm^–1^. Importantly, in the absence of lifetime broadening one would have expected to observe well-resolved transitions to the various levels of such modes.^35^ The observation that such is not the case indicates that the observed spectrum is indeed subject to extensive lifetime broadening.

Focusing for the moment on the onset of the two transitions, we observe that the width of the experimentally observed band at 32,560 cm^–1^ is larger (roughly a factor of 2–3) than predicted theoretically for the two transitions. One could therefore speculate that this band actually consists of overlapping S_2_(ππ*) ← S_0_ and S_3_(ππ*) ← S_0_ transitions, but it is clear that with the present data, it is not possible to go beyond the level of speculation. The Franck–Condon spectrum predicted for the S_2_(ππ*) ← S_0_ transition is further characterized by vibrational activity in the ∼700 and ∼1650 cm^–1^ regions, while for the S_3_(ππ*) ← S_0_ transition such activity is dispersed over a much larger region. In this respect, the experimentally observed spectrum would appear to favor an assignment to the S_2_(ππ*) excitation spectrum, although we also directly notice that the relative intensities of the pertaining vibrational regions are not as large as predicted by the calculations. We, therefore, conclude that the experimentally observed excitation spectrum is in agreement with what is to be expected on account of the planarization of the benzopyrone and phenyl moieties upon excitation but that further theoretical studies are needed to come to a quantitative understanding of the further details of this spectrum.

**Figure 3:**
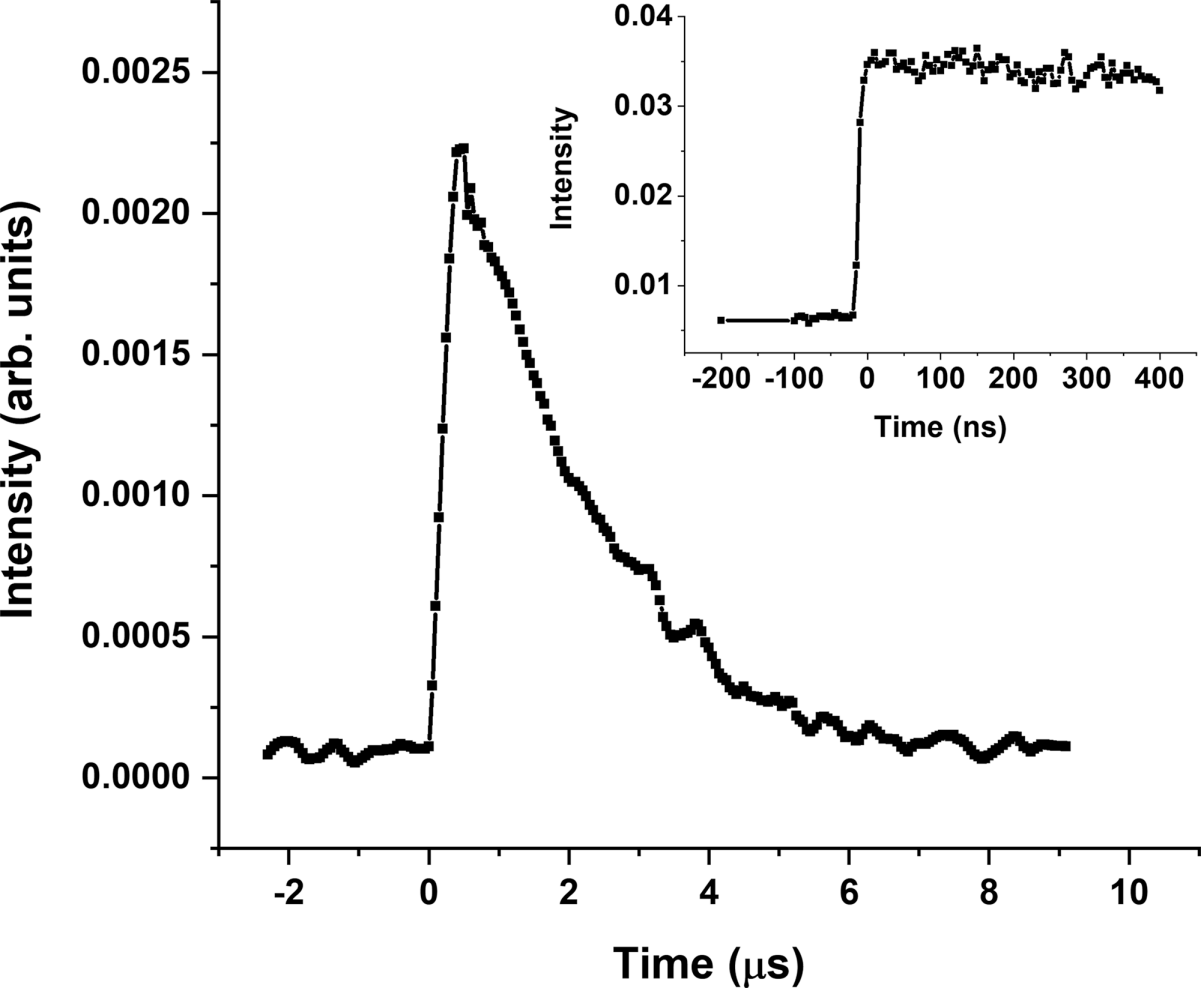
Pump–probe traces of the ion yield obtained after excitation at 32,563 cm^–1^ with the insert displaying a blowup of the initial 400 ns time region.

Pump–probe traces of the ion yield obtained after excitation at 32,563 cm^–1^—the maximum of the band observed in Figure 2—show initially a constant signal followed by decay on a microsecond timescale that can be attributed to the fact that molecules that have been excited in the molecular beam travel away from the spot where ionization takes place (Figure 3). Similar traces are observed for different excitation energies (see Supporting Information SI2). The trace thus actually indicates that after excitation molecules are ionized from a state that does not decay on the timescale during which they are “visible” to the ionization laser. Such a conclusion is in line with solution experiments in which an almost near-unit triplet formation is observed upon UV radiation.^29^ We, therefore, conclude that in our experiments we observe ions that have been generated by ionization from triplet states. A further observation that can be made is that the trace does not show a fast decay for near-zero-time delays between excitation and ionization lasers followed by an apparent slower decay caused by the removal of excited molecules from the ionization spot. On the timescale of the present experiments fast internal conversion of the initially excited ππ* state to the nπ* state will have taken place. The absence of a fast decay thus indicates that for time-overlapping excitation and ionization lasers, the contribution to the signal of ions associated with ionization from the excited nπ* singlet state is negligible. We, therefore, also conclude that intersystem crossing from the excited singlet manifold to the triplet manifold occurs at rates that are at least one or two orders of magnitude larger than the inverse of the pulse durations of the excitation and ionization lasers. Such a conclusion nicely supports the results of calculations on intersystem crossing rates, which predict rates on the order of 10^11^ s^–1^ for flavone under isolated conditions.^28,46^

**Figure 4:**
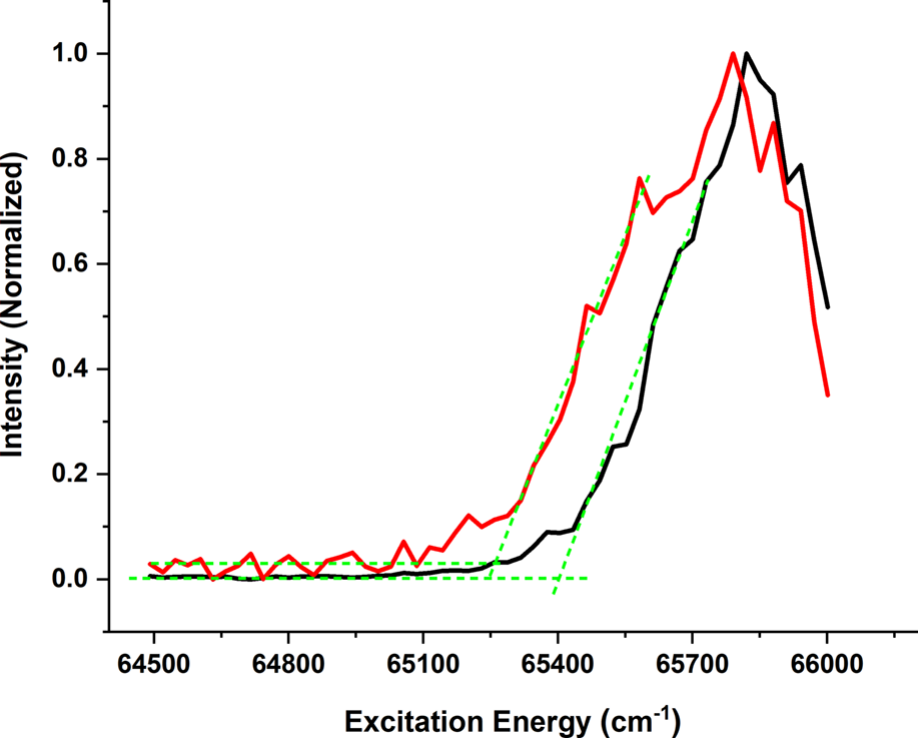
Photoionization spectrum obtained for excitation of flavone at 31,158 cm^–1^ using ion detection (red trace) and electron detection (black trace).

The excitation spectrum of flavone is of importance for assessing its properties as a UV absorber and, related to that, its potential applications in sunscreen formulations. As discussed in the introduction, flavonoids are also well known for their antioxidant properties for which detailed knowledge of their ionization energies is important. In the first instance, we have tried to perform zero-kinetic-energy pulsed-field-ionization (ZEKE-PFI) experiments via the observed S_2_(ππ*) ← S_0_ band. However, such experiments were not successful, most probably due to rapid electronic and vibrational autoionization processes.^47^ We, therefore, reverted to measuring photoionization spectra to determine the adiabatic ionization threshold. One-color photoionization was in this case not possible since the wavelengths that would need to be used are in the region of Figure 2, and thus lead to spectra that are not uniquely determined by ionization thresholds but also by resonance enhancement. We have therefore chosen to employ a two-color scheme in which two-photon ionization occurs via absorption of a photon with a fixed energy of 31,158 cm^–1^—which is in the very tail of the band observed in the excitation spectrum of Figure 2—and absorption of a photon with an energy that is scanned. The photoionization spectrum recorded in this way using ion detection is depicted in Figure 4 as the red trace. From this curve, an ionization threshold of 65,259 ± 30 cm^–1^ is obtained, which yields an adiabatic ionization energy of 65,424 ± 30 cm^–1^ (8.112 ± 0.004 eV) when extrapolated to zero electric field.^47,48^ A further determination and confirmation of this energy have been obtained using electron detection for which much lower electric fields are used. The ionization threshold spectrum obtained in this way is shown as the black trace in Figure 4. As expected, this spectrum is systematically shifted to higher energies compared to the red trace, giving rise to an ionization threshold of 65,415 ± 30 cm^–1^ (8.109 ± 0.004 eV), which is in good agreement with the value obtained with ion detection. Photoionization spectra recorded at several other excitation wavelengths (see Supporting Information SI3) lead to the same threshold and show that this threshold can indeed be associated with the adiabatic ionization energy and not with a threshold to a vibrationally excited cation. Previous He(I) photoelectron studies have reported a vertical ionization energy of 8.52 ± 0.2 eV^33^ implying an internal reorganization energy upon ionization of 0.41 eV, which is in line with what is expected. These numbers also compare favorably with our calculations, which predict adiabatic and vertical ionization energies to the ground ionic state of 8.18 and 8.40 eV, respectively.

**Figure 5:**
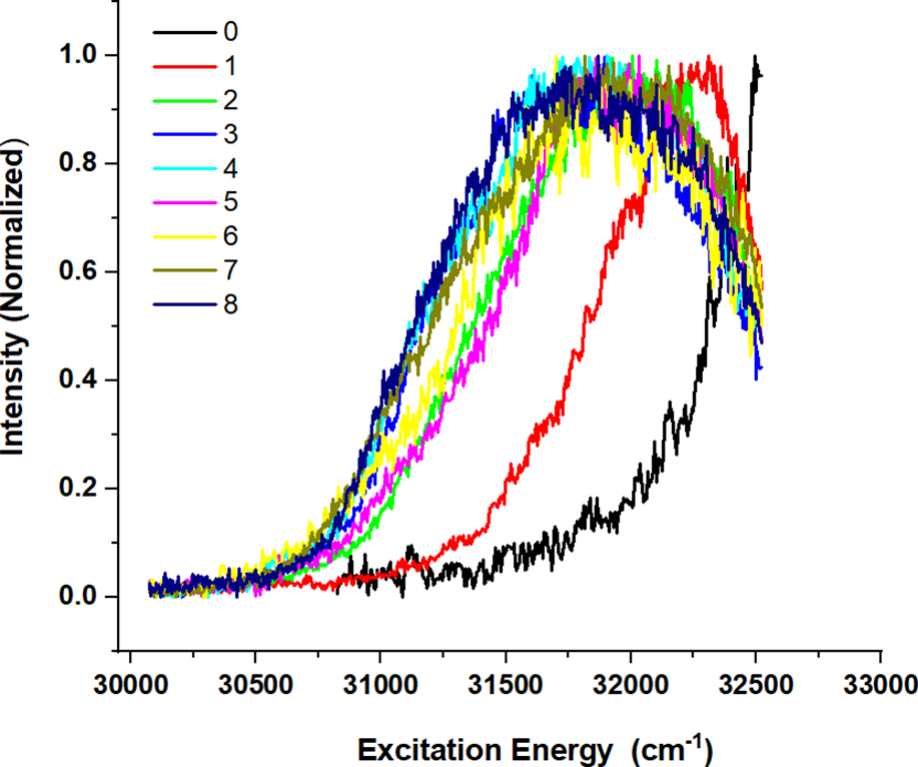
(1 + 1′) R2PI excitation spectrum of flavone–(H_2_O)_*n*_ clusters obtained by monitoring ions at the mass of the molecular ion. Values of *n* are indicated in the inset.

Under biological conditions flavone is not isolated but subject to interactions with solvent molecules. From this point of view, it is of interest to determine how the spectroscopic properties of flavone as determined above are modified by a solvent. To this purpose, we have performed microsolvation studies in which we study flavone complexed with an increasing number of water molecules. Figure 5 reports (1 + 1′) R2PI excitation spectra recorded for flavone–(H_2_O)_*n*_ complexes with up to eight water molecules (see Supporting Information SI4 for a further discussion of the possible role of dissociative ionization). These spectra show that the addition of one water molecule leads to a redshift of the spectrum of ∼300 cm^–1^. A similar shift (∼300 cm^–1^) results from the addition of a second water molecule, but it has to be noticed that the spectrum at the same time significantly broadens. Interestingly, further addition of water molecules leads to significantly smaller redshifts of the spectrum and no further broadening of the spectrum. Apparently, conformational heterogeneity starts to be of influence for two water molecules and more, but its effects on the excitation spectrum are minor once two water molecules have been added. The observed redshifts are in line with the general notion that ππ* states shift to lower energies in polar solvents. Similarly, one would expect the lower-lying nπ* state to be shifted to higher excitation energies upon increasing microsolvation, but in the spectra that have been recorded here, there is no indication of the presence of the S_1_(nπ*) state within the recorded excitation energy range.

Quantum chemical calculations show as expected that the lowest-energy structure of the flavone–H_2_O complex arises from complexation at the carbonyl site. In addition, and in agreement with our expectations, these calculations find that further addition of water molecules preferably occurs by binding to the already complexed water molecules as this affords strong hydrogen bonds. In this respect, it is interesting that the addition of a second water molecule leads to a similar redshift of the flavone–H_2_O spectrum as the redshift observed in going from flavone to flavone–H_2_O since the second water molecule is not directly attached to the aromatic system of flavone. Apparently, delocalization of the π orbitals does not extend beyond the second water molecule or is considered from another point of view, the charge reorganization occurring upon forming a hydrogen bond between water and the carbonyl oxygen is influenced by the hydrogen bond formed between the two water molecules in flavone–(H_2_O)_2_ but not by a third or more water molecule.

Pump–probe experiments on microsolvated flavone show that complexation with water does not lead to a discernible influence on decay dynamics. In all cases, long-lived species are observed whose R2PI signal disappears because the excited complexes travel away from the excitation spot. Although one would expect that under solvated conditions the intersystem crossing rate will decrease because of the increasing energy gap between the S_1_(nπ*) and T_1_(ππ*) states in polar solvents, the present pump–probe experiments do not give evidence for such a decrease nor for a change in intersystem crossing yield. We thus conclude that under the intrinsic excited state decay dynamics of flavone are retained under the microsolvated conditions explored here.

## Conclusions

The present study has provided detailed insight into the spectroscopic and dynamic properties of flavone, a key chromophore in providing protection from harmful UV radiation as well as from harmful radicals. Our molecular beam studies have shown that previously obtained excitation spectra are not associated with the strongly absorbing S_2_(ππ*) state but involve valence or Rydberg states at the two-photon level. The one-photon excitation spectrum recorded here shows in contrast primarily a very broadband with minor discernible vibrational resolution. Quantum chemical calculations confirm that the absence of resolved vibrational bands can very well be attributed to extensive Franck–Condon activity of low-frequency normal modes activated because of planarization of the phenyl and pyrone planes upon excitation, in combination with lifetime broadening due to internal conversion from the initially excited bright S_2_(ππ*) state to the dark S_1_(nπ*) state. Pump–probe studies of the R2PI signal find that the observed ion signal is associated with ionization from an electronically excited state that is instantaneously populated on the timescale of our experiments and that has a lifetime longer than can be probed in our experiments. Such conclusions are in agreement with the generally accepted decay path of the S_2_(ππ*) state, which involves ISC to the triplet manifold with near-unity quantum yield.

Microsolvation studies in which flavone is increasingly complexed with water molecules demonstrate that the addition of one and two molecules results in significant redshifts of the excitation spectrum as would indeed be expected for a ππ* state but that further complexation has limited influence on both the maximum and width of the absorption band. On the accessible timescales of the present experiments, the observed decay dynamics, on the other hand, do not appear to be influenced by microsolvation.

Finally, our studies have also contributed to the further knowledge of the antioxidant properties of flavone. These properties are closely related to its ionization energy. The present study has enabled a much more accurate determination of the adiabatic ionization threshold than possible up till now [65,415 ± 30 cm^–1^ (8.110 ± 0.004 eV)].

## Supporting Information Available

Experimental details; additional pump–probe R2PI traces of flavone; additional two-color photoionization spectra; role of dissociative ionization of flavone–(H_2_O)_*n*_ clusters; and equilibrium (TD-)DFT geometries of flavone in S_0_, S_1_, S_2_, and S_3_ at the B3LYP/TZVP and wB97XD/cc-pVDZ levels of theory (PDF)
