# Supplementary material for: Resonance-Enhanced Multiphoton Ionization Studies of the Lower Electronically Excited States of Flavone
Source: J Phys Chem A. 2023 Feb 13;127(7):1649–55. doi: 10.1021/acs.jpca.3c00202 (PMC9969512; doi:10.1021/acs.jpca.3c00202)
Supplement: Supplementary file 1 — jp3c00202_si_001.pdf [file jp3c00202_si_001.pdf]

Supporting Information

Resonance Enhanced MultiPhoton Ionization Studies of the Lower Electronically Excited States of Flavone

Jiayun Fan[a] *and Wybren Jan Buma*[*a,b]

[a] Van 't Hoff Institute for Molecular Sciences, University of Amsterdam, Science Park 904, 1098 XH Amsterdam, The Netherlands

[b]Radboud University, Institute for Molecules and Materials, FELIX Laboratory, Toernooiveld 7c, 6525 ED Nijmegen, The Netherlands.

E-mail: w.j.buma@uva.nl

SI1. Experimental details

Resonant two-photon ionization (R2PI) spectroscopy in combination with mass-resolved ion detection or photoelectron detection has been performed in a setup designed to perform laser spectroscopy on molecules cooled in a supersonic free jet expansion. In this setup the molecular beam is created with an injector assembly that consists of a stainless-steel oven in which a glass container with the sample is placed and kept in the experiments on flavone at a temperature of 150 °C. The oven is connected to a pulsed valve (General Valve Iota One) equipped with a 0.5 mm diameter nozzle that is typically kept 5 °C above the oven temperature to prevent clogging of the nozzle. Typically, a pulse duration of 180-220 μs is used. In the present experiments neon at a backing pressure of 1.5 bar is led through the oven and expanded through the nozzle into the vacuum chamber. After passing through a skimmer with a diameter of 2 mm, the molecular beam is introduced into the ion source region. Here either mass-resolved ion detection is performed using a reflectron type time-of-flight spectrometer (R.M. Jordan Co.) or electron detection using a custom-built setup (R.M. Jordan Co.) similar to the one described in ref.[1,2] In the case of mass-resolved ion detection typically an extraction field of 752 V/cm was used, while for electron detection a much lower field of 2.36 V/cm was employed. Typical pressures with the molecular beam running are $2 \cdot 10^{-5}$ mbar for the source chamber, $5 \cdot 10^{-7}$ mbar for the ionization chamber and $2 \cdot 10^{-7}$ mbar for the detection chamber. Without the molecular beam typical pressures are $2 \cdot 10^{-7}$ mbar, $5 \cdot 10^{-8}$ mbar and $4 \cdot 10^{-8}$ mbar in the source, ionization and detection chamber, respectively. Under such conditions and for molecules of the size of flavone typically rotational temperatures of 2-10 K and vibrational temperatures around 20 K can be expected.[3]

SI2. Pump-probe R2PI traces of flavone

In the main article the pump-probe trace has been shown that was obtained at the maximum of the broad band observed in the R2PI excitation spectrum that covers the 31500-33500 cm^{-1} region. Figure S1 show pump-probe traces at different positions in this band. In all cases initially a constant signal is observed followed by a decay on the microsecond timescale that can be attributed to the travelling away of excited molecules from the excitation spot. Under the employed experimental conditions of a neon expansion with a speed of 786 m/s and an excitation spot with a diameter of about 3 mm one expects the signal to disappear after about 4 μs, as is indeed observed.

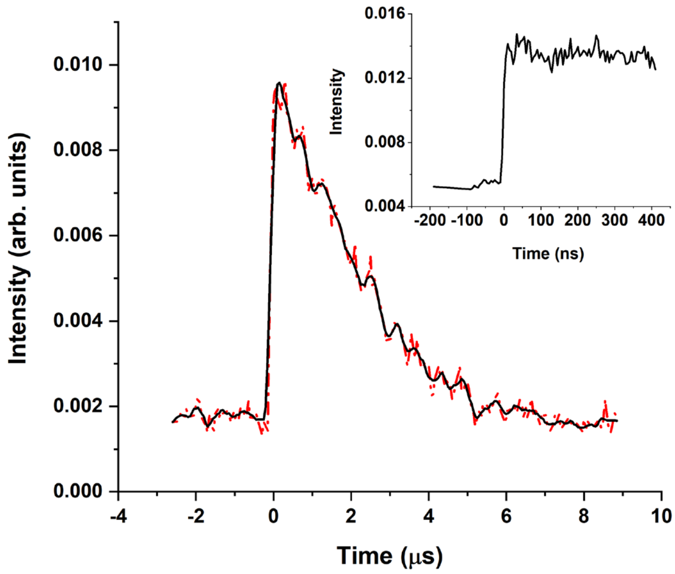

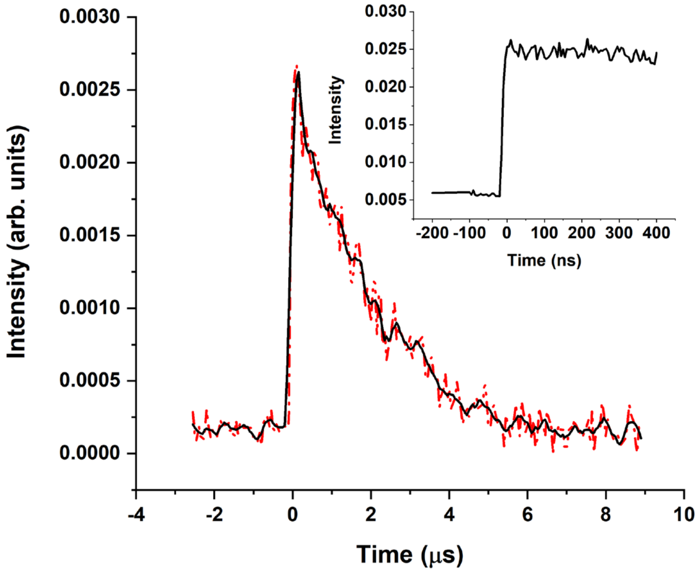

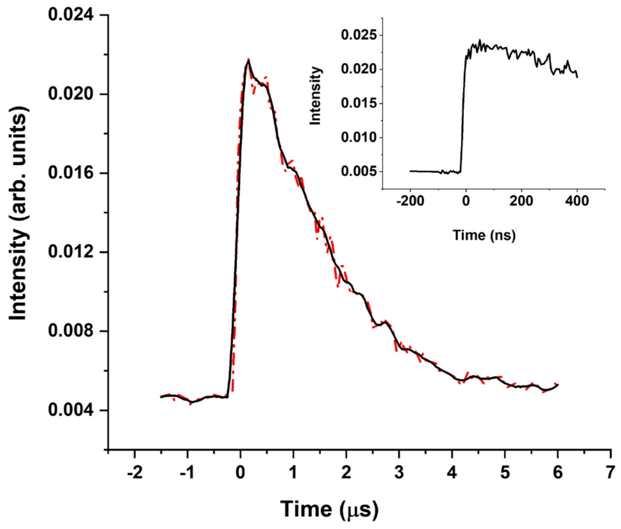

Figure S1. Pump-probe traces of the ion yield obtained after excitation at 32000 (top), 32258 (middle) and 32787 (bottom) cm^{-1} with the insert displaying a blow-up of the initial 400 ns time region.

SI3. Two-color photoionization spectra

In the main article the two-color photoionization spectrum after excitation at 31158 cm^{-1} has been shown in Figure 4. Figure S2 shows similar spectra for excitation at several other wavelengths. In these cases an extraction field of 515 V/cm was employed as compared to the fieldd of 752 V/cm employed for recording Figure 3 of the main article. Extrapolation to zero electric field yields ionization thresholds of 65444±30, 65441±30 and 65463±30 cm^{-1} for excitation at 31496 (top), 31746 (middle), and 32000 (bottom) cm^{-1} which reproduces that threshold reported in the main article. We thus conclude that this threshold can indeed be associated with the adiabatic ionization energy of flavone and not with the threshold to a vibrationally-excited cation.

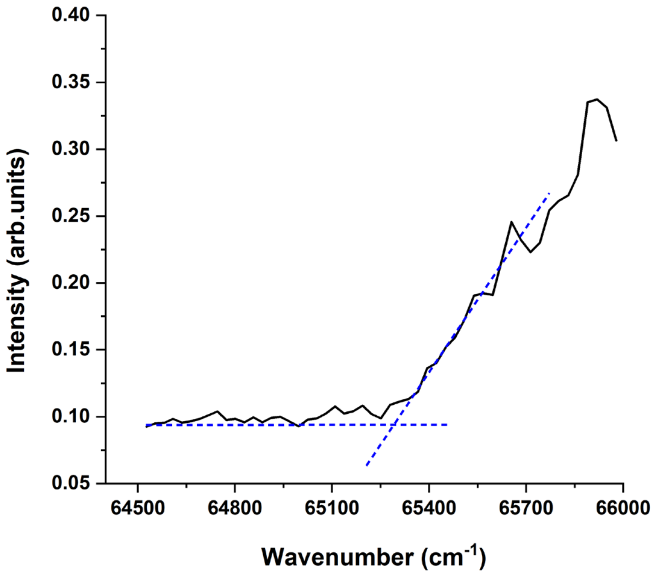

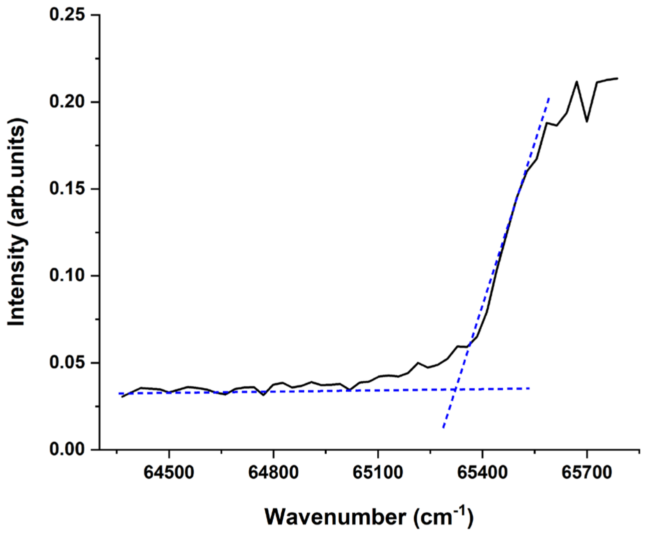

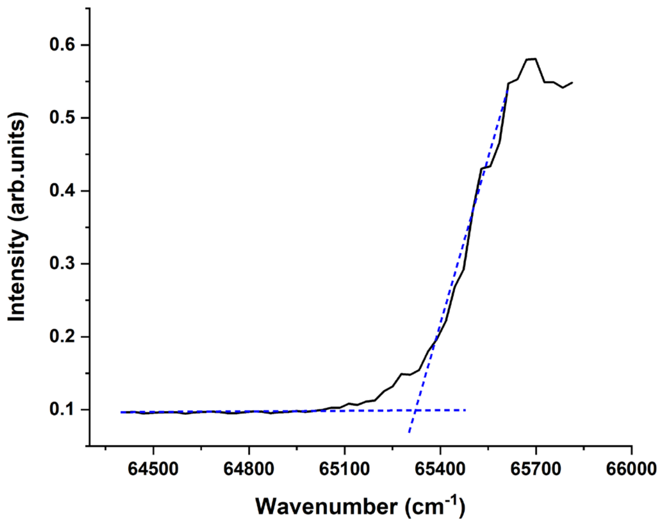

Figure S2. Ionization threshold spectra obtained for excitation of flavone at 31496 (top), 31746 (middle), and 32000 (bottom) cm^{-1} and using ion detection.

SI4. Role of dissociative ionization of flavone-$(H_2O)_n$ clusters

The cluster of flavone-$(H_2O)_n$ could in principle contribute to the ion signal detected at the mass of clusters with a lower number of water molecules if (flavone-$(H_2O)_n)^+$ would dissociate into (flavone-$(H_2O)_{n-m})^+$ + $(H_2O)_m$. However, since resonance enhancement of the ion signal takes place at the one-photon level, one would then observe resonance enhancement occurring by absorption of the photon by the flavone-$(H_2O)_n$ cluster in these lower mass channels. Figure 5 in the main article shows that certainly for the flavone-$(H_2O)_{n=1-3}$ channels no 'bleeding-through' is occurring. For larger clusters this is less clear although the differences in onset and widths do suggest that dissociative ionization plays at most a minor role, if at all.

SI5. Equilibrium (TD-)DFT geometries of flavone in S_0, S_1, S_2, and S_3 at the B3LYP/TZVP and wB97XD/cc-pVDZ levels of theory

S_0 (B3LYP/TZVP) Energy = -728.101953 Hartree

C	4.31042900	-0.69548700	0.05859600
C	3.60869900	0.49096800	-0.03858800
C	2.20819200	0.49237300	-0.03210000
C	1.54087100	-0.72949500	0.07192300
C	2.23570400	-1.93404100	0.17245800
C	3.61908800	-1.90887300	0.16435700
H	5.39291100	-0.69012800	0.05362600
H	4.11159000	1.44593900	-0.12125500
H	1.68331600	-2.86070000	0.25568100
H	4.16852200	-2.83886500	0.24173800
C	-0.57735000	0.32455500	-0.01257600
C	-0.01144500	1.54743100	-0.13315200
H	-0.63271400	2.42308200	-0.24782500
O	0.17180400	-0.80814800	0.08871500
O	1.95488700	2.84996000	-0.22675200
C	1.42873100	1.74636000	-0.13635200
C	-2.02293700	0.04079500	0.00462400
C	-2.94194400	1.02914500	0.37923900
C	-2.50261300	-1.22315100	-0.36078300
C	-4.30384400	0.76344700	0.37402400
H	-2.59053000	2.00198800	0.69662200
C	-3.86679900	-1.48366100	-0.36656300
H	-1.80307000	-1.99579000	-0.64735900
C	-4.77207100	-0.49274800	-0.00129200
H	-5.00114700	1.53643600	0.67221300
H	-4.22335300	-2.46377600	-0.65834800
H	-5.83531800	-0.69840300	-0.00370100

S_1 (B3LYP/TZVP) Energy = -727.989309 Hartree

C	4.31980400	-0.68181600	0.00000100
C	3.61178500	0.51146000	-0.00000300
C	2.20913900	0.49755500	-0.00001100
C	1.53676500	-0.73677700	-0.00000300
C	2.24793600	-1.93013700	-0.00000200
C	3.63864500	-1.89726500	0.00000600
H	5.40186200	-0.66534900	0.00000600
H	4.13287000	1.45866300	-0.00000600
H	1.70149800	-2.86362600	0.00000100
H	4.19107600	-2.82825100	0.00000900
C	-0.60967200	0.30968100	-0.00000600
C	0.01056700	1.56355200	0.00000300
H	-0.59571900	2.45461000	0.00001600
O	0.17981600	-0.81867500	-0.00001400
O	2.00254800	2.84419600	0.00001500
C	1.38222300	1.69120900	0.00000100
C	-2.01434100	0.04592800	-0.00000200
C	-2.96933300	1.09413300	-0.00000800
C	-2.50624600	-1.28412100	0.00000700
C	-4.32414200	0.82240300	-0.00000500
H	-2.64749300	2.12662200	-0.00001900
C	-3.86601800	-1.53837800	0.00001000
H	-1.80696600	-2.10731800	0.00001200
C	-4.79072600	-0.49405000	0.00000400
H	-5.02901800	1.64518800	-0.00001000
H	-4.21204600	-2.56523100	0.00001700
H	-5.85328700	-0.69973500	0.00000600

S_2 (B3LYP/TZVP) Energy = -727.967958 Hartree

C	4.29929700	-0.73835700	0.00005700
C	3.57351600	0.48953300	0.00004100
C	2.20151400	0.50769500	0.00001800
C	1.51981700	-0.72597700	-0.00002100
C	2.24055800	-1.96553600	-0.00001000
C	3.62151100	-1.95614100	0.00002300
H	5.38098200	-0.71501200	0.00008600
H	4.09048000	1.44169700	0.00005300
H	1.67191800	-2.88553200	-0.00003700
H	4.17043000	-2.88793800	0.00003300
C	-0.63342500	0.38607400	-0.00003100
C	0.00699600	1.61841900	-0.00005600
H	-0.59957500	2.51142200	-0.00007800
O	0.21102800	-0.80931600	-0.00005800
O	2.02318900	2.87135700	-0.00002400
C	1.41190600	1.79399100	-0.00002100
C	-2.00489700	0.06437000	-0.00000700
C	-2.99620900	1.09506000	0.00004200
C	-2.47482000	-1.28486600	-0.00003200
C	-4.33834100	0.78814900	0.00005900
H	-2.69519700	2.13383800	0.00007000
C	-3.82704400	-1.56557400	-0.00001300
H	-1.76457100	-2.09799800	-0.00006900
C	-4.78015200	-0.54315000	0.00003100
H	-5.06278200	1.59430300	0.00009600
H	-4.14946100	-2.60052000	-0.00003400
H	-5.83732300	-0.77272800	0.00004500

S_3 (B3LYP/TZVP) Energy = -727.952512 Hartree

C	4.30939300	-0.67541000	0.00000700
C	3.59340200	0.50973900	-0.00000200
C	2.19403600	0.49905200	-0.00000100
C	1.52729700	-0.75594400	-0.00000500
C	2.24123300	-1.95167600	0.00000900
C	3.62914300	-1.90953800	0.00001000
H	5.39177500	-0.66051400	0.00000600
H	4.07992600	1.47620400	-0.00000100
H	1.70153500	-2.88924400	0.00001000
H	4.19137000	-2.83488700	0.00001800
C	-0.60696100	0.30524300	-0.00001400
C	0.00474300	1.56219200	-0.00001000
H	-0.61596900	2.44622300	-0.00001500
O	0.17199900	-0.85898100	-0.00002000
O	1.97789400	2.87260900	0.00000900
C	1.41801000	1.76424600	-0.00000100
C	-1.99584000	0.04168800	-0.00000600
C	-2.96248700	1.11116300	0.00000200
C	-2.49708900	-1.30593100	-0.00000800
C	-4.30835500	0.83653500	0.00001200
H	-2.62639000	2.13812600	-0.00000100
C	-3.85004700	-1.54996200	0.00000100
H	-1.79141900	-2.12300100	-0.00001500
C	-4.77380300	-0.48973800	0.00001200
H	-5.01974500	1.65316400	0.00001900
H	-4.20926200	-2.57178300	0.00000000
H	-5.83700700	-0.69326300	0.00001800

S_0 (wB97XD/cc-pVDZ) Energy = -727.8744937 Hartree

C	4.30627900	-0.70491900	0.05723500
C	3.60644900	0.48608100	-0.03397600
C	2.20434100	0.48988200	-0.02849800
C	1.52961600	-0.72798400	0.06894300
C	2.22145100	-1.93857800	0.16263100
C	3.60748600	-1.91791600	0.15575100
H	5.39700500	-0.70388200	0.05312400
H	4.11070300	1.45025300	-0.11252200
H	1.65920500	-2.86930700	0.24049600
H	4.15662200	-2.85823300	0.22845800
C	-0.57007800	0.33102200	-0.01033100
C	-0.00959800	1.55821000	-0.12424100
H	-0.63638400	2.44120100	-0.23311300
O	0.16654700	-0.80162500	0.08437500
O	1.97301100	2.84730200	-0.21979300
C	1.43671500	1.75335300	-0.13064100
C	-2.02137500	0.04609600	0.00474300
C	-2.94308300	1.04008800	0.35943000
C	-2.49244200	-1.22723900	-0.34063800
C	-4.30741900	0.76931100	0.35271200
H	-2.59293600	2.02739400	0.66311600
C	-3.85862400	-1.49369900	-0.34785200
H	-1.78142100	-2.00725400	-0.61089700
C	-4.77002200	-0.49719500	-0.00347400
H	-5.01368000	1.55114600	0.63625700
H	-4.21301300	-2.48777400	-0.62533900
H	-5.84075200	-0.70804200	-0.00699300

S_1 (wB97XD/cc-pVDZ) Energy = -727.7386635 Hartree

C	4.32170100	-0.70066500	-0.00000300
C	3.61918900	0.50299400	0.00000200
C	2.21478600	0.50173800	0.00000100
C	1.53435700	-0.73323800	-0.00000300
C	2.23577300	-1.92898300	-0.00000800
C	3.63387300	-1.91082400	-0.00000700
H	5.41232600	-0.68927800	-0.00000200
H	4.15087200	1.45412400	0.00000600
H	1.67541700	-2.86413600	-0.00001200
H	4.18106900	-2.85443300	-0.00001100
C	-0.59052300	0.32967100	0.00000000
C	-0.00019400	1.56461400	0.00000700
H	-0.60946200	2.46417800	0.00001200
O	0.17238100	-0.80975900	-0.00000500
O	1.98758000	2.86588200	0.00001000
C	1.40416300	1.69943700	0.00000500
C	-2.02589400	0.05537100	0.00000100
C	-2.97731800	1.09243400	-0.00001600
C	-2.48908100	-1.27241300	0.00001600
C	-4.33682300	0.80861000	-0.00001600
H	-2.66008900	2.13561800	-0.00003100
C	-3.85263600	-1.54851100	0.00001500
H	-1.76807600	-2.08813300	0.00002700
C	-4.78529600	-0.51322000	0.00000000
H	-5.05535900	1.63008300	-0.00003000
H	-4.18868800	-2.58705600	0.00002700
H	-5.85416300	-0.73205100	-0.00000100

S_2 (wB97XD/cc-pVDZ) Energy = -727.7130685 Hartree

C	4.32680100	-0.67261400	-0.00000500
C	3.60185500	0.51767500	-0.00000900
C	2.20490000	0.50069900	-0.00000600
C	1.55126900	-0.73830500	0.00000100
C	2.25690100	-1.93567000	0.00000800
C	3.65296700	-1.89430200	0.00000500
H	5.41722300	-0.64933200	-0.00000900
H	4.09022600	1.49319400	-0.00001500
H	1.71100400	-2.87931800	0.00001500
H	4.21399400	-2.83021300	0.00000900
C	-0.61164900	0.24810900	0.00001100
C	-0.00680600	1.55356500	0.00001000
H	-0.63654300	2.44057100	0.00002000
O	0.18081900	-0.84069000	-0.00000100
O	1.94572800	2.87246200	-0.00000900
C	1.41439200	1.75264600	-0.00000300
C	-1.99985700	0.00371300	0.00001100
C	-2.93656500	1.09899600	0.00001200
C	-2.52670000	-1.33097200	0.00000200
C	-4.29794900	0.85190900	0.00000000
H	-2.57959500	2.12814800	0.00002600
C	-3.88509900	-1.54360200	-0.00001200
H	-1.83430800	-2.17179500	0.00000700
C	-4.78874400	-0.45723800	-0.00001400
H	-4.99394900	1.69257500	0.00000400
H	-4.27059900	-2.56471600	-0.00002200
H	-5.86413600	-0.64094600	-0.00002700

S_3 (wB97XD/cc-pVDZ) Energy = -727.7031777 Hartree

C	4.28126700	-0.71354900	0.00002500
C	3.58257000	0.49303800	0.00001300
C	2.18702000	0.50251100	-0.00000300
C	1.49696200	-0.75175100	-0.00000800
C	2.20531200	-1.97580700	0.00000500
C	3.58886300	-1.94872500	0.00002100
H	5.37242300	-0.71167500	0.00003700
H	4.08394600	1.46100400	0.00001600
H	1.64002300	-2.90743800	0.00000300
H	4.14974800	-2.88413300	0.00003200
C	-0.60312100	0.35681100	-0.00001800
C	0.01795400	1.59837400	-0.00001800
H	-0.59889200	2.49582800	-0.00001500
O	0.17349500	-0.83009100	-0.00002700
O	2.03339900	2.86447400	-0.00001300
C	1.43295400	1.78261400	-0.00001300
C	-1.99344800	0.06774100	-0.00000800
C	-2.97168500	1.11583800	0.00002300
C	-2.46737200	-1.28551900	-0.00002700
C	-4.31746600	0.81806600	0.00003600
H	-2.65006400	2.15688400	0.00003800
C	-3.82277700	-1.55697900	-0.00001400
H	-1.74378500	-2.09828100	-0.00005100
C	-4.76195100	-0.51673800	0.00001800
H	-5.04647400	1.63025700	0.00006000
H	-4.16242600	-2.59427700	-0.00002900
H	-5.83013500	-0.73877500	0.00003000

References

(1) Smolarek, S. UV and IR laser spectroscopy of isolated molecular structural dynamics. *Ph.D. thesis*, University of Amsterdam, **2011**.

(2) Wang, K.; Rodham, D. A.; McKoy, V.; Blake, G. A. High-resolution zero-kinetic-energy pulsed field ionization photoelectron spectra of the Na(H_2O) complex. *J. Chem. Phys.* **1998**, *108*, 4817-4827.

(3) Tan, E. M.; Amirjalayer, S.; Smolarek, S.; Vdovin, A.; Zerbetto, F.; Buma, W. J. Fast photodynamics of azobenzene probed by scanning excited-state potential energy surfaces using slow spectroscopy. *Nat. Commun.* **2015**, *6*, 1-7.
